# Location of pedicle screw hold in relation to bone quality and loads

**DOI:** 10.3389/fbioe.2022.953119

**Published:** 2022-09-02

**Authors:** Frédéric Cornaz, Mazda Farshad, Jonas Widmer

**Affiliations:** ^1^ Department of Orthopedics, Balgrist University Hospital, Zurich, Switzerland; ^2^ Institute for Biomechanics, ETH Zurich, Zurich, Switzerland

**Keywords:** pedicle screw, lumbar spine, instrumentation, primary stability, bone density, screw bone interface

## Abstract

**Introduction**: Sufficient screw hold is an indispensable requirement for successful spinal fusion, but pedicle screw loosening is a highly prevalent burden. The aim of this study was to quantify the contribution of the pedicle and corpus region in relation to bone quality and loading amplitude of pedicle screws with traditional trajectories.

**Methods**: After CT examination to classify bone quality, 14 pedicle screws were inserted into seven L5. Subsequently, Micro-CT images were acquired to analyze the screw’s location and the vertebrae were split in the midsagittal plane and horizontally along the screw’s axis to allow imprint tests with 6 mm long sections of the pedicle screws in a caudal direction perpendicular to the screw’s surface. Force-displacement curves in combination with the micro-CT data were used to reconstruct the resistance of the pedicle and corpus region at different loading amplitudes.

**Results**: Bone quality was classified as normal in three specimens, as moderate in two and as bad in two specimens, resulting in six, four, and four pedicle screws per group. The screw length in the pedicle region in relation to the inserted screw length was measured at an average of 63%, 62%, and 52% for the three groups, respectively. At a calculated 100 N axial load acting on the whole pedicle screw, the pedicle region contributed an average of 55%, 58%, and 58% resistance for the normal, moderate, and bad bone quality specimens, respectively. With 500 N load, these values were measured at 59%, 63%, and 73% and with 1000 N load, they were quantified at 71%, 75%, and 81%.

**Conclusion**: At lower loading amplitudes, the contribution of the pedicle and corpus region on pedicle screw hold are largely balanced and independent of bone quality. With increasing loading amplitudes, the contribution of the pedicle region increases disproportionally, and this increase is even more pronounced in situations with reduced bone quality. These results demonstrate the importance of the pedicle region for screw hold, especially for reduced bone quality.

## Introduction

Dorsal pedicle screw instrumentation is one of the main techniques used for spinal fusion and spinal stabilization (Martin et al., 2019; Reisener et al., 2020). Pedicle screw based dorsal instrumentation systems have been developed into a versatile and effective method, and can be combined with a multitude of other implants, such as intervertebral cages, crosslinks, or laminar bands to achieve the aspired construct characteristics. While substantial progress has been achieved in the last few decades, insufficient screw hold and screw loosening still pose a major complication in the clinical routine in up to 50% of patients (Kim et al., 2020), which can result in pain, loss of reduction, or neurologic deficit (Ohlin et al., 1994; Röllinghoff et al., 2010; El Saman et al., 2013; Mac-Thiong et al., 2013; Glennie et al., 2015; Bredow et al., 2016; Ohba et al., 2019). Most of the time, revision surgery is the consequence.

To reduce the rate of pedicle screw loosening, alternative trajectories have been proposed, such as the cortical bone trajectory (CBT) (Santoni et al., 2009; Glennie et al., 2015) or screw augmentation methods using bone cement (Burval et al., 2007; El Saman et al., 2013; Wang et al., 2019). However, CBT screw loosening remains a relevant problem with a similar prevalence (Santoni et al., 2009; Glennie et al., 2015; Patel et al., 2016; Elmekaty et al., 2018) and bone cement augmentation poses an additional complication risk, such as cement extrusion or embolism (Becker et al., 2008; Janssen et al., 2017; Guozhi et al., 2019). More fundamental optimizing principles have been well adopted, such as maximizing screw length and diameter (Matsukawa et al., 2016), while other approaches have not been graded to outweigh the potential drawbacks in most clinical situations, such as bicortical screw placement (Spirig et al., 2021) or crosslink-augmentation (Cornaz et al., 2021).

**FIGURE 1:**
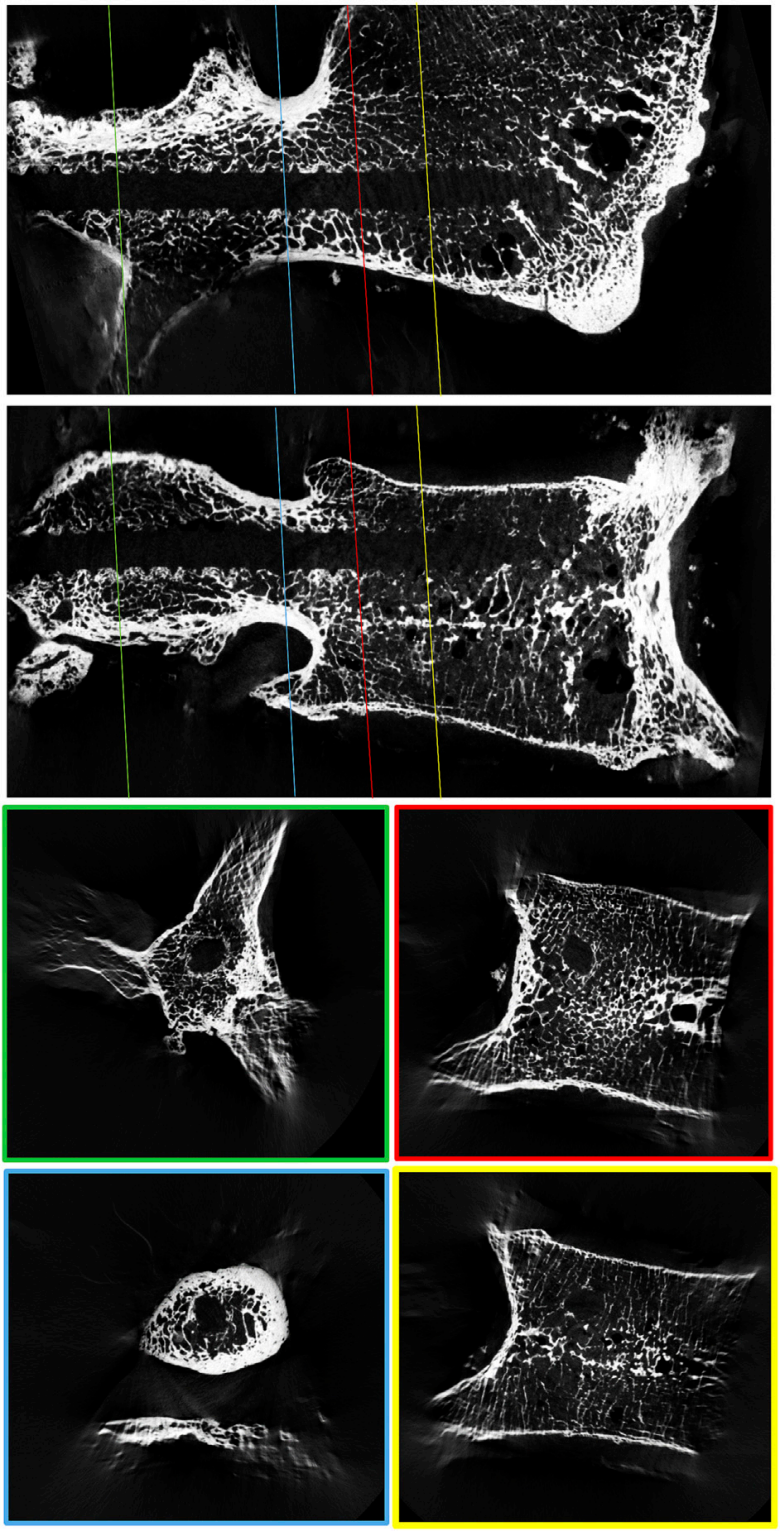
μCT reconstruction of a lumbar vertebral body to visualize the spatial variability in trabecular bone density around a 3D-printed replica of a pedicle screw.

To quantify screw hold, axial load-to-failure pullout tests (Burval et al., 2007; Aichmair et al., 2017; Lai et al., 2018), different cyclic toggling test setups simulating more physiological loading conditions (Weiser et al., 2017; Liebsch et al., 2018; Spirig et al., 2021), as well as finite element simulations (Biswas et al., 2018; Chevalier et al., 2018; Van den Abbeele et al., 2018; Widmer et al., 2020; Biswas et al., 2022) have been employed to guide surgeons, researchers, and the industry into the direction of optimized screw fixation strength. While different aspects of screw hold can be analyzed with these methods, they usually attempt to investigate the behavior of the bone-screw interaction at a “global” level. However, it appears essential to lay an additional focus on the regional distribution because large spatial differences of screw hold must be expected due to anatomical and microstructural differences along the screw’s trajectory (Figure 1). This perspective could help us to understand the bone-screw interaction and guide innovation successfully towards optimized implant geometries or more effective screw trajectories.

The aim of this study was to qualitatively analyze the local support along traditional pedicle screw trajectories and to quantify the contribution of the pedicle and corpus region in relation to bone quality and loading amplitude.

## Materials and methods

### Specimen preparation

Seven L5 vertebrae of fresh frozen human cadavers were used for this study after approval by the local ethics authorities (BASEC Nr. 2017–00874). Clinical CT-scans (SOMATOM Edge Plus, Siemens Medical Solutions, Erlangen, Germany) were acquired to exclude fractures and to identify anatomical particularities, such as lumbosacral transitional anomalies. The acquisition parameters included a slice thickness of 1 mm, pixel dimensions of 0.3516 × 0.3516 mm, a peak voltage of 90–140 kVp, an x-ray tube current of 34–72 mA, and exposure times of 1,000–1,232 ms. The convolution kernel Br60s was used. To correct for the effect of different peak voltages, the grey values were approximately corrected to values corresponding to 120 kVp by using the reference values provided by the work of Afifi et al. (2020). Following the method described by Schreiber et al., the mean Hounsfield unit (HU) value of three axial elliptical regions of interest (inferior to the superior end plate, in the middle of the vertebral body and superior to the inferior end plate) was measured in the corrected CT reconstructions. The bone quality of the specimens with a mean HU value of 120 HU and larger was categorized as normal, between 90 and 119 HU as moderate (osteopenic) and below 90 HU as bad (osteoporotic) according to the reported distributions in the analyzed cohort (Schreiber et al., 2011).

After thawing, the vertebrae were skeletonized, and the pedicle screw entry points were prepared with a bone rongeur (Vaccaro et al., 2020). The pedicles were prepared with a Lenke bone probe, and the maximal insertion depth for the pedicle screw was measured. Instrumentation was performed with commercially available self-tapping polyaxial pedicle screws with a standardized diameter of 5 mm and length of 55 mm (MUST Pedicle Screw System, Medacta International SA, Castel San Pietro, Switzerland). The screws were inserted to the predefined insertion depth and the screw’s rotation was brought to one of the two predefined rotational positions, to guarantee identical thread imprints at the screw’s tip in all specimens. The pedicle screw was then removed, and a 3D-printed replica of the screw with a modified tail was inserted to omit metal artifacts, to improve contrast in the following micro-CT scans, and to provide mechanical reference for the screw trajectory. The 3D-printed screws were made from medical grade polyamide (P2200) and were printed using selective laser sintering (SLS) technology (P395, EOS e-Manufacturing Solutions, Munich, Germany). Insertion of the 3D-printed screw was performed with only minimal torque, preventing additional damage to the vertebral bodies. The vertebral bodies were cut in the midsagittal plane to meet the dimension limits of the specimen holder of the micro-CT scanner.

### μCT imaging

μCT scans of all 14 specimens were acquired (Bruker, SkyScan 1176; PANalytical’s Microfocus Tube, Source Voltage = 90 kV; Source Current = 278 μA) with a voxel size of 35.43 μm. The picture planes of the reconstructions were oriented to align with the screw’s axis using dedicated software (Skyscan 1176 control software) to guarantee the same orientation of the specimens in both the µCT-scans and later biomechanical testing.

### Biomechanical testing

**FIGURE 2:**
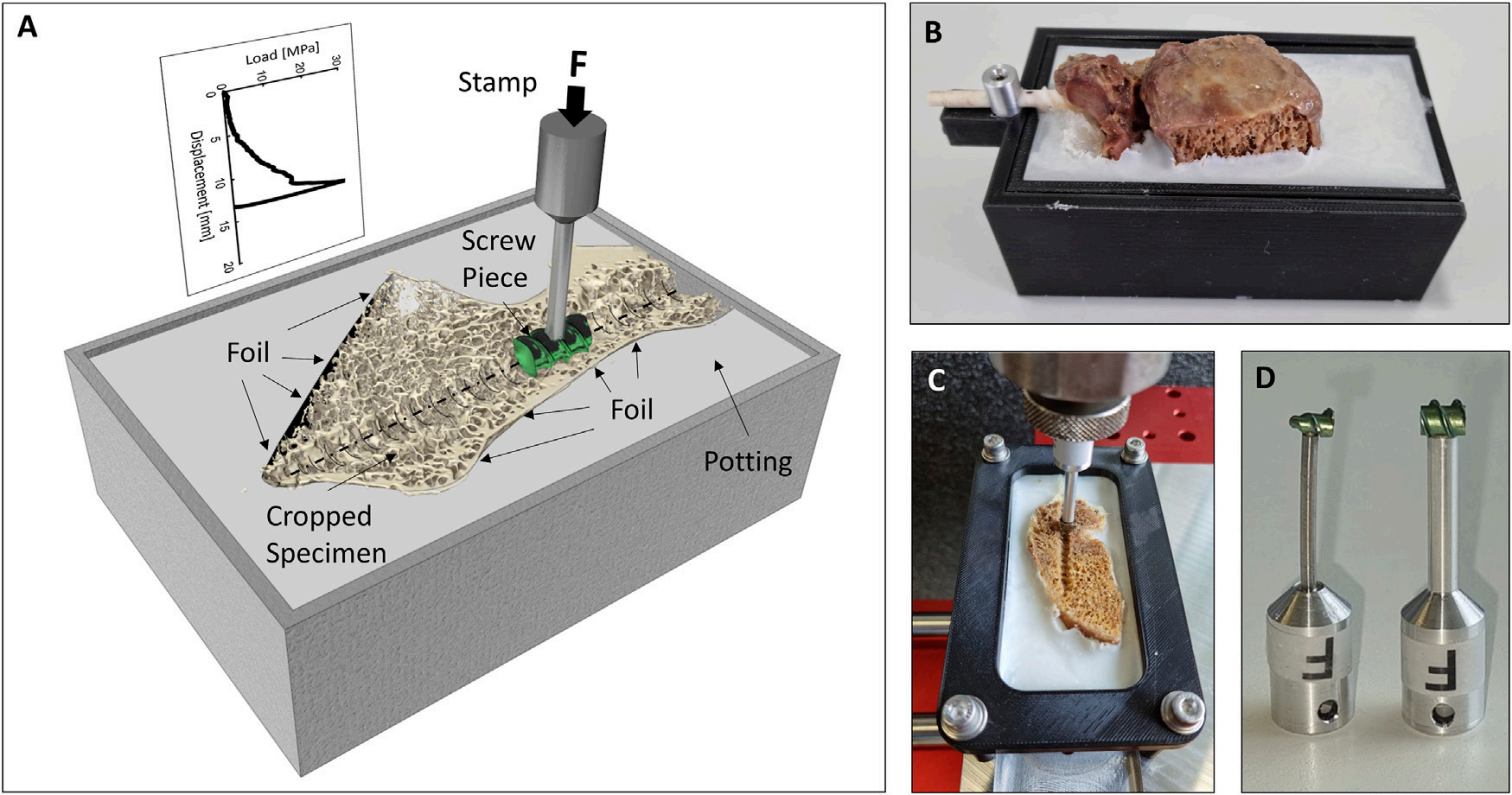
**(A)** Illustration of the biomechanical testing method. **(B)** Specimen after potting with the holding apparatus and the 3D-printed pedicle screw still in place. **(C)** Cropped specimen just prior to testing. **(D)** Photography of the tip (left) and center (right) screw imprint probes.

To achieve reliable specimen fixation and adequate load distribution over a large surface area during testing, Polymethyl methacrylate (PMMA) potting (Beracryl D28 and SCS-Beracryl Monomer, Suter Kunststoffe AG, Fraubrunnen, Switzerland) was used. After wrapping the specimens in plastic foil to prevent potting intrusion (Pfeiffer et al., 1996), the tail of the 3D-printed screw replica was used by a holding apparatus to position the specimen above the potting box with the screw’s trajectory horizontal and central, and with the midsagittal cut being vertical (Figure 2B). PMMA was poured to the upper rim of the potting box. After curing, the holding apparatus and the 3D-printed screws were removed, and the cranial aspect of the specimens was carefully cut with a bandsaw just cranial to the center of the pedicle screw axis (parallel to the undersurface of the potting box). With that method, the caudal screw imprint was made available for biomechanical testing (Figures 2A,C). To measure the resistance of the vertebral body perpendicular to the screw axis at different positions along the screw’s trajectory, a pedicle screw of the same type as used for instrumentation was sectioned into pieces of 6 mm length with an offcut of 6 mm starting from the screw’s tip. With the thread pitch of this type of pedicle screw being 3 mm, the center pieces were identical, and therefore only one center piece was required. The sections of the pedicle screws were fixed to a mounting pin to be used in the biomechanical test setup (Figure 2D). A static testing machine (Zwick/Roell Allroundline 10kN, ZwickRoell GmbH & Co. KG, Germany) was equipped with a mechanical setup, which allowed for the fixation of the potting box in the center axis of the machine, and which also allowed to move the box along the screw axis by increments of 12 mm between tests. Complementing the use of the placeholder screw as a positioning reference, the location of the threads of the screw piece was visually controlled to match the imprint in the vertebrae. After validation of the position, a punch imprint was performed at a constant rate of 0.2 mm/s, until a maximum load of 350 N for the tip piece or 500 N for the center piece was obtained. These maximal load values were chosen to guarantee overloading of the trabecular bone, while preventing failure of the cortical bone. Additional abortion criteria to protect the specimen and setup were a drop in maximal load of more than 50% or an insertion depth of more than 24 mm. The testing sequence (e.g., tip to tail) was reversed for half of the specimens to limit any systematic error due to potential effects on the adjacent testing location. Testing was performed at room temperature and the specimens were frequently strayed with phosphate buffered saline (PBS) to help prevent dehydration. To compensate for any deformation of the mechanical test setup or any motion between specimen and potting, reference measurements were conducted with the imprint stamps pushing on an aluminum plate, which has been placed on top of the specimen to allow for load distribution and to prevent any failure. The load-deflection curves from these reference measurements were used to correct the imprint measurements. Deformation of the whole setup including the sample was below 0.7 mm for a load application of 500 N.

### Data analysis

The projected surface area of the two imprint probes (Figure 2D) was measured with a telecentric camera system (Edmund Optics #62–921, 182 mm WD, 0.28X, Edmund Optics Inc., Barrington, NJ, United States). This information about the projected surface area was used to convert the applied forces to stress values. The following analysis is based on the stress-displacement curves, which allow to compensate for the difference in surface area of the tip piece compared to the center piece. The parasagittal reconstructions of µCT-scans (along the screw trajectories) were used to measure the insertion length of the pedicle screw in the corpus and the pedicle region. The corpus region was defined to begin at the position of the first line perpendicular to the screw’s trajectory, which intersects with the caudal endplate region and does not have contact with the posterior cortical bone. The projected surface area of the pedicle screw in the two regions was calculated.

**FIGURE 3:**
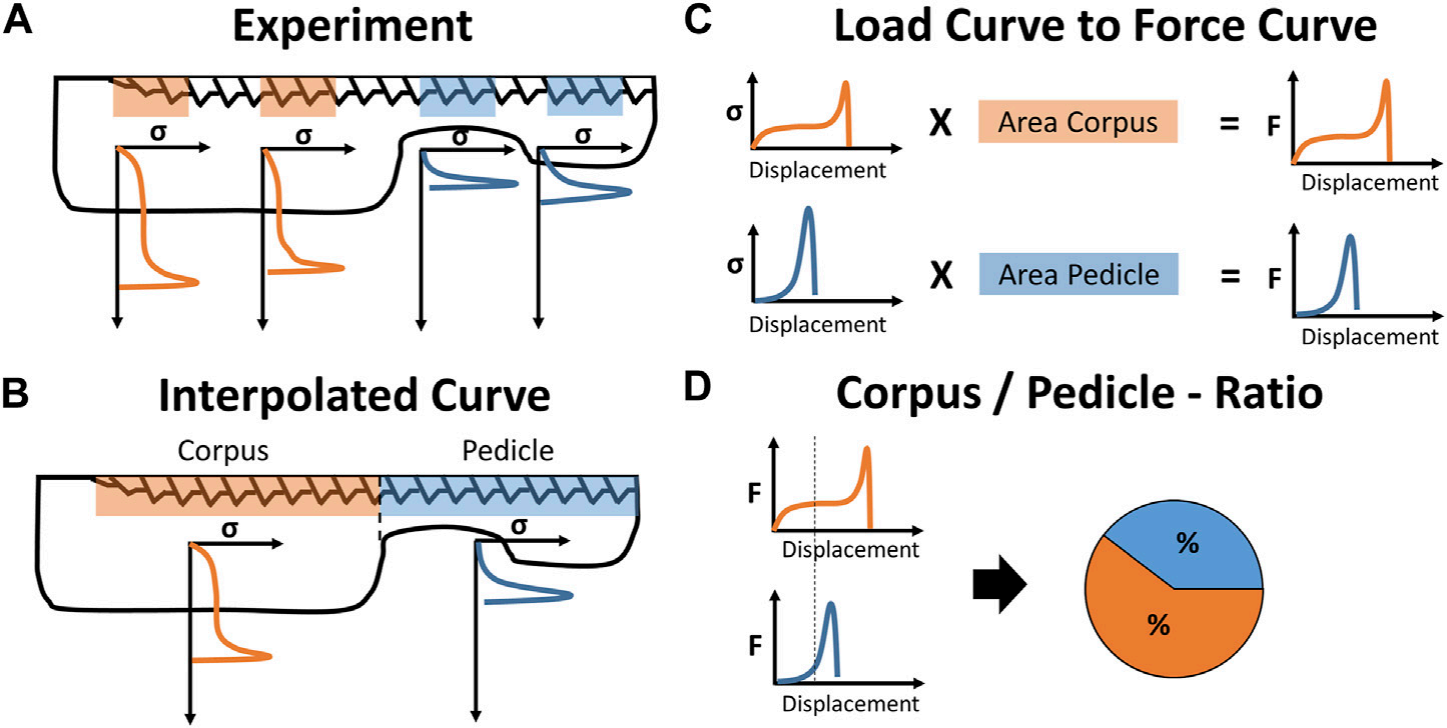
Illustration of data processing: **(A)** The experimentally derived stress-displacement curves are assigned to the corpus and pedicle region and **(B)** interpolated to a single stress-displacement curve for either anatomical region. **(C)** The projected screw surface area of both regions is used to convert the stress-values to force-values and **(D)** displacement-controlled virtual screw imprint tests are performed to calculate the load distribution between the corpus and pedicle region for specific loading conditions.

The screw-imprint measurements were only assigned to the corpus region when they were fully localized in the corpus region (Figure 3A). The missing imprint data between the experimentally measured locations were interpolated with the neighboring stress-displacement curves of the same anatomical (Figure 3B). For sectors between two measurements of the same anatomical region, the average of these measurements was used. For a region with only one neighboring measurement of the same anatomical region, the averaged stress-deflection curve of the region was used. With that, the local stress-deflection curves along the whole screw were defined. With the known surface area of these sections, the local resisting force for any screw displacement (in the direction of the performed imprint tests) could be calculated (Figure 3C).

Assuming an average strength of 2.4 MPa (Banse et al., 2002) and a pedicle screw with a projected surface area of around 200 mm^2^ (screw diameter of 5 mm and implanted screw length of 40 mm), the screw-bone interface would fail at a total loading amplitude of around 480 N. With the variability in trabecular bone strength ranging from 0.6 to 7.8 MPa (Mosekilde and Mosekilde, 1986; Banse et al., 2002), forces of 120–1560 N could be resisted with the same pedicle screw. Therefore, total loading amplitudes of 100 N, 500 N, and 1,000 N were chosen to represent loading conditions without expected overloading (100 N), with partial overloading (500 N), and with local overloading in most cases (1000 N). The virtual screw imprint tests simulated a caudally directed displacement of the whole pedicle screw until the above-mentioned forces (100 N, 500 N, and 1,000 N) were countered by the available bone surface under the pedicle screw (Figure 3D). This method allowed to compute the screw displacement, the relative force contribution of the corpus and pedicle region, and the mean stresses at the bone surface in these two regions. These values were computed for every screw and were pooled for the three bone quality categories.

## Results

**FIGURE 4:**
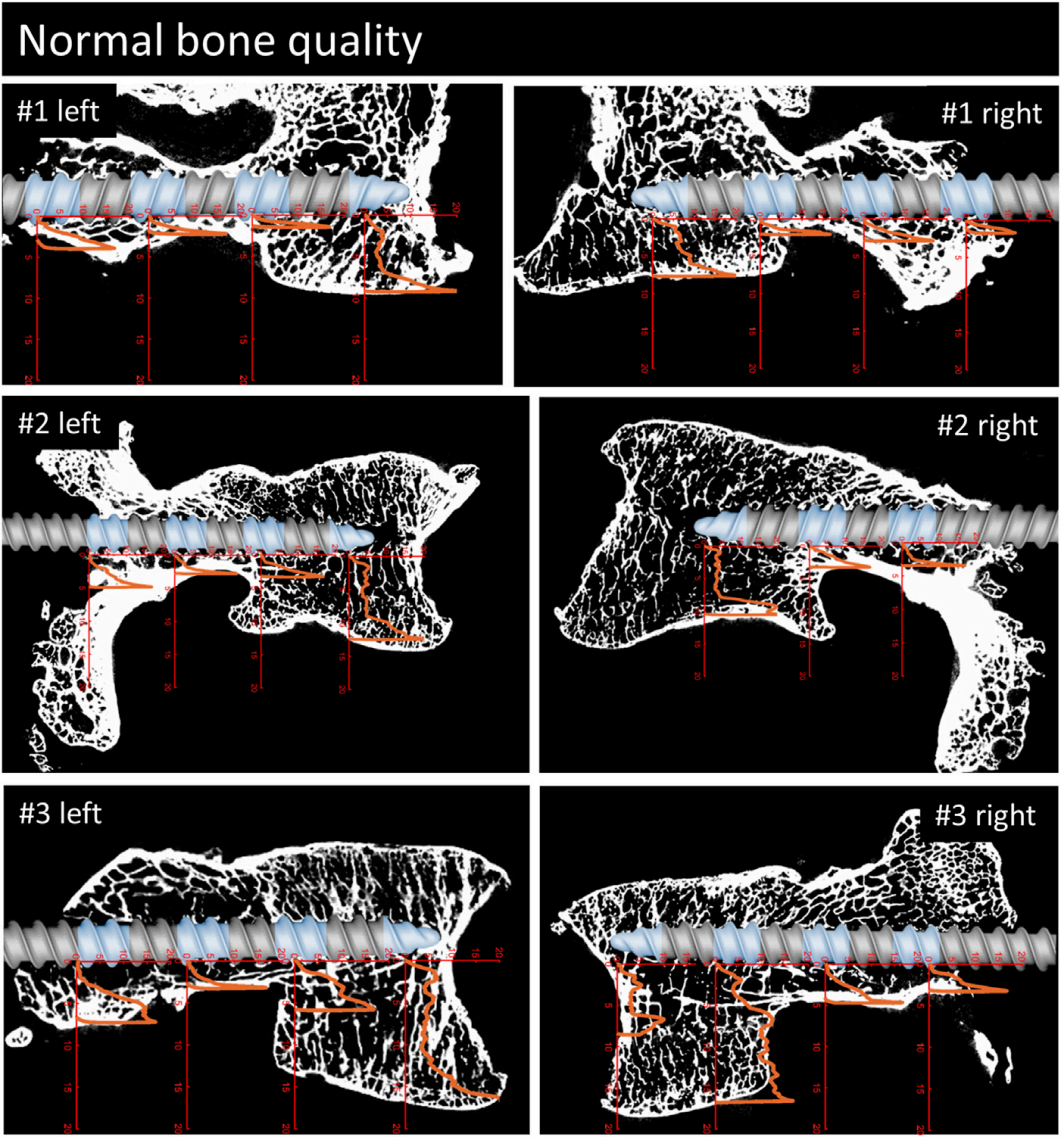
Parasagittal reconstruction of the micro-CT scans of the vertebrae with normal bone quality. An image of the pedicle screw is overlaid graphically to illustrate the position of the screw and the measurement locations (blue sections). The load [MPa]-displacement [mm] curves are depicted in orange.

The demographic characteristics of the specimens are listed in Table 1. The specimens are ordered with decreasing bone density and the same ordering is used throughout the manuscript. Bone quality was classified as normal in three specimens, as moderate in two, and as bad in two specimens, resulting in six, four, and four pedicle screws per group. Of the seven L5 vertebrae, one showed lumbosacral transitional anomaly (specimen #1, Figure 4). Because of the irregular shape of the caudal aspect of the vertebral body, the imprint tests were conducted in the cranial instead of the caudal direction for this specimen. During the imprint test of the tip piece of specimen #3 on the right-hand side (Figure 4), the test had to be stopped manually due to progressive lateral-deviation of the imprint probe. The imprint test at the most posterior testing location of specimen #1 on the right-hand side was performed with the protocol of the tip piece instead of the center piece, resulting in an imprint test with a maximum load of 350 N instead of 500 N. Analysis of the micro-CT data did not reveal any bone damage due to the insertion of the 3D-printed screw replica. All of the data was included in the evaluation.

**TABLE 1:** Demographic characteristics of the specimens.

Donor #	Bone quality	Sex	Age	Height [cm]	Weight [kg]	BMI
1	Normal	Male	69	188	176.9	50
2	Normal	Male	69	183	98.9	29.6
3	Normal	Male	66	183	95.3	28.4
4	Moderate	Male	62	168	54.4	19.4
5	Moderate	Male	64	180	64.8	36.1
6	Bad	Female	59	165	50.3	18.5
7	Bad	Female	57	175	68	22.1
Mean			63.7	177.4	86.9	29.2
Standard deviation			4.3	7.8	40.6	10.3

The projected surface area of the 6 mm long screw tip piece was measured at 16.42 mm^2^ and the 6 mm long center piece was measured at 29.79 mm^2^. The applied maximum loads (350 and 500 N, respectively) correspond to a maximal stress of 21.3 MPa for the tip piece and 16.8 MPa for the center piece.

**FIGURE 5:**
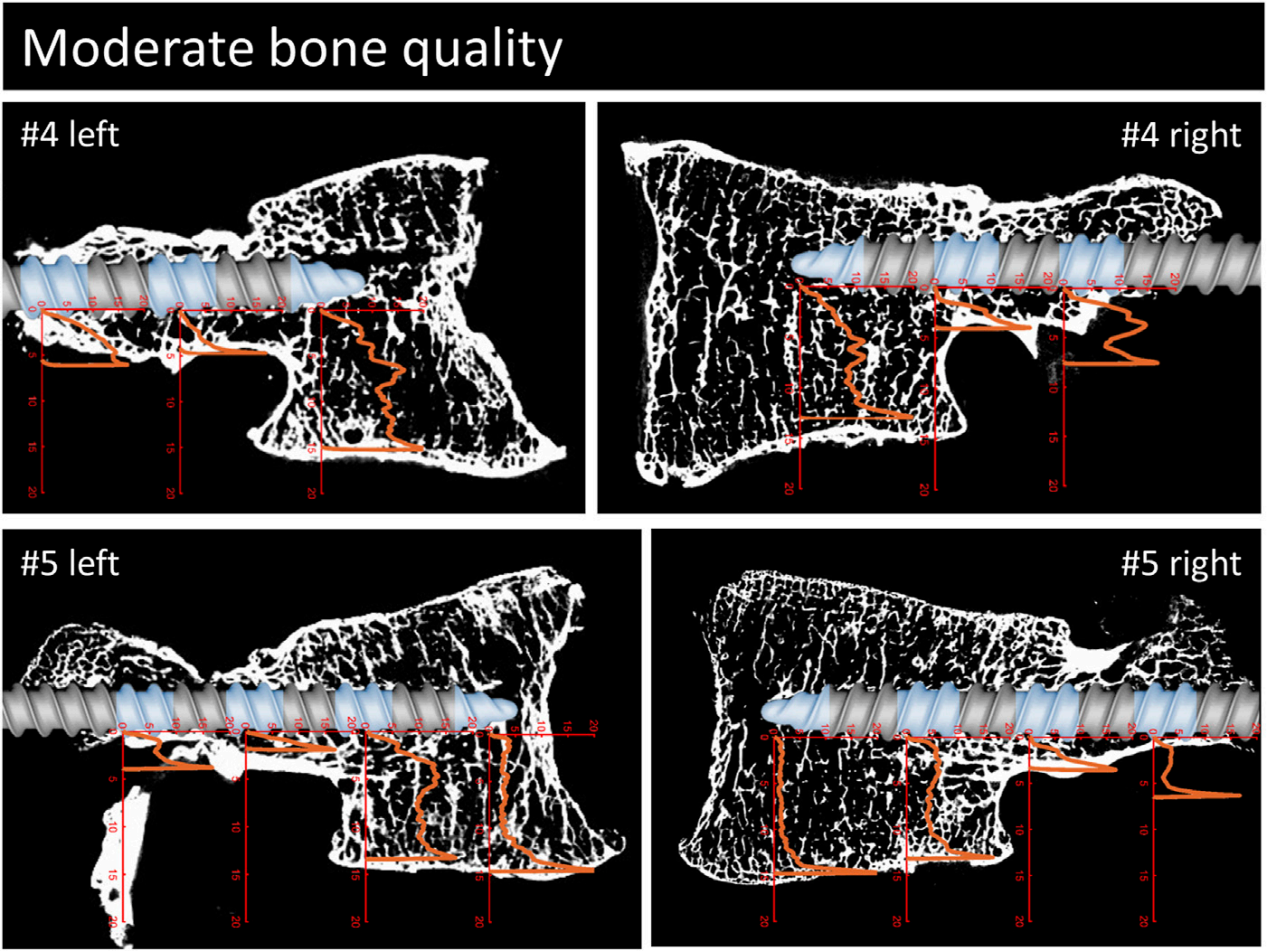
Continuation of Figure 3. Parasagittal reconstruction of the micro-CT scans of the vertebrae with moderate bone quality. An image of the pedicle screw is overlaid graphically to illustrate the position of the screw and the measurement locations (blue sections). The load [MPa]-displacement [mm] curves are depicted in orange.

**FIGURE 6:**
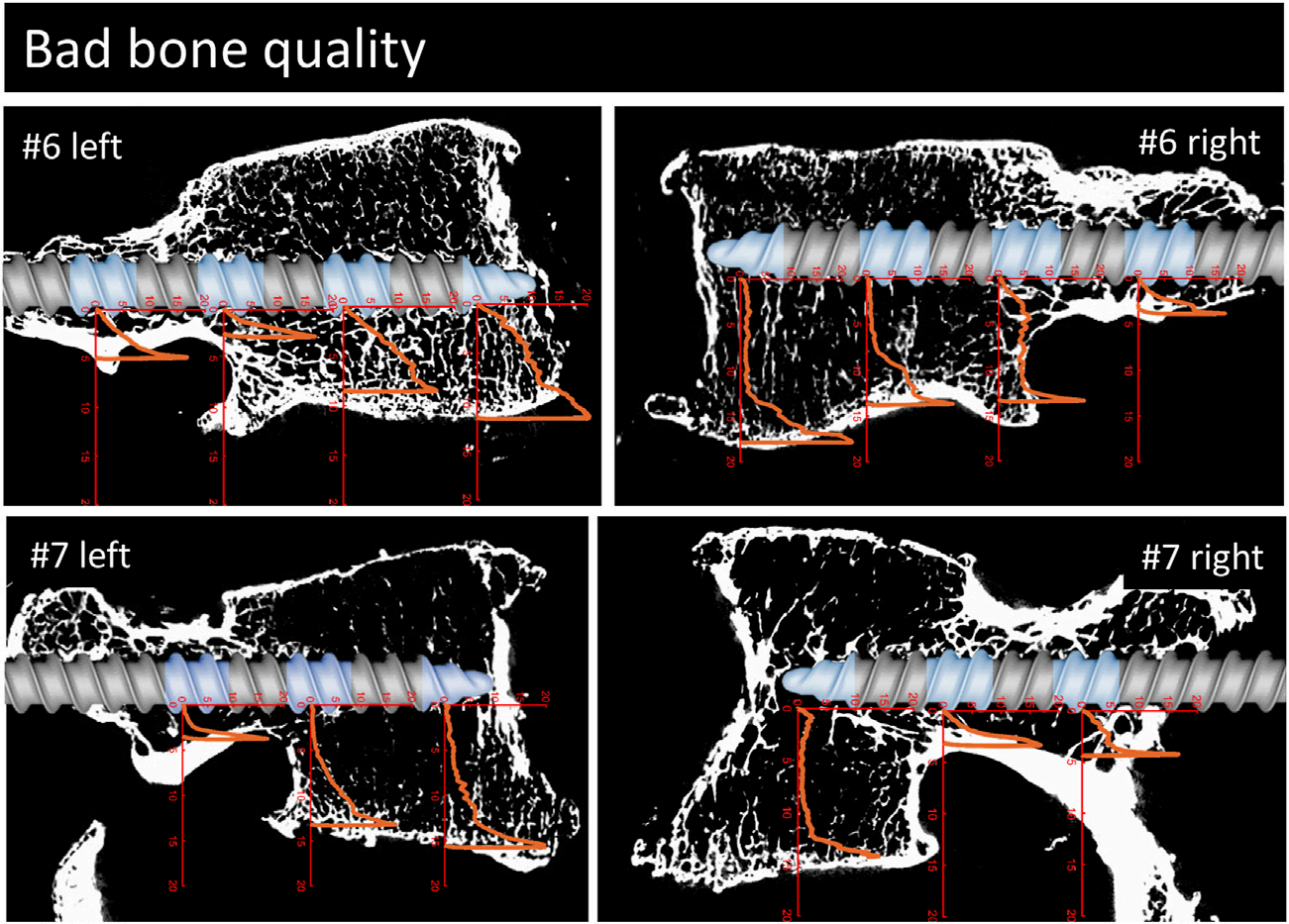
Continuation of Figures 3, 4. Parasagittal reconstruction of the micro-CT scans of the vertebrae with bad bone quality. An image of the pedicle screw is overlaid graphically to illustrate the position of the screw and the measurement locations (blue sections). The load [MPa]-displacement [mm] curves are depicted in orange.

For a qualitative analysis of the results, the parasagittal micro-CT scans in plane with the screw trajectories are plotted in Figures 4–6 with an overlay of the pedicle screw and the stress-displacement curves at every measured location.

**FIGURE 7:**
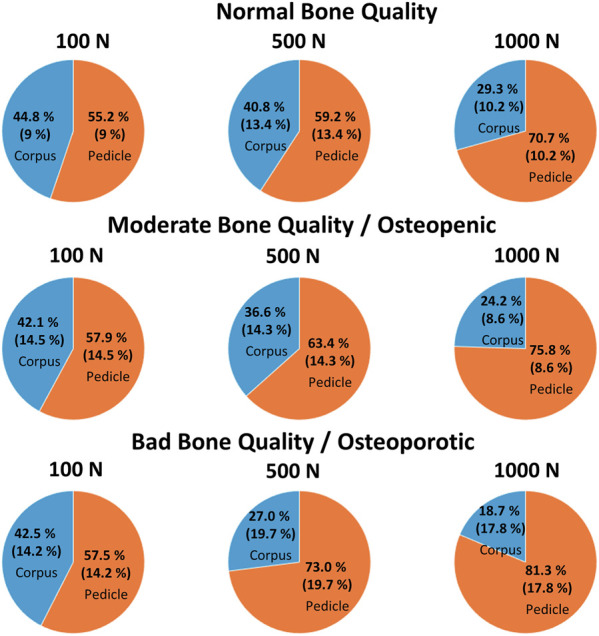
The mean (and the standard deviation) of the relative contribution of the pedicle and corpus region for normal, moderate, and bad bone quality for a total of 100 N, 500 N, and 1,000 N acting on the pedicle screw, which are illustrated using pie plots.

The implanted screw length was measured at an average of 42 mm, 36 mm, and 41 mm for the group with normal, moderate, and bad bone quality. The screw length in the pedicle region compared to the total implanted screw length was measured at averages of 63%, 62%, and 52%, which relates to an average of 68%, 68%, and 55% of the projected screw area being localized in the pedicle region for the three bone quality groups, respectively (Table 2). At a calculated 100 N axial load acting on the whole pedicle screw, the pedicle region contributed an average of 55%, 58%, and 58% resistance for the normal, moderate, and bad bone quality specimens, respectively. When this contribution is set into relation with the projected screw’s surface in the pedicle region, the resistance in the pedicle region is 0.81, 0.85, and 1.04 times the resistance in the corpus region (normalization to projected surface area, Table 2). With 500 N load, the average absolute contribution of the pedicle region was 59%, 63%, and 73%, relating to a relative contribution of the pedicle region of 0.87, 0.93, and 1.33 times compared to the corpus region. With 1000 N load, the contribution of the pedicle region was quantified at 71%, 75%, and 81%, relating to the pedicle region providing 1.04, 1.13, and 1.49 times more resistance per area than the corpus region for normal, moderate, and bad bone quality, respectively. The relative contribution of the pedicle and corpus region in dependence of loading amplitude and bone quality are visualized in Figure 7. The average displacement of the screw to counter the calculated force of 100 N was 0.24 mm, 0.28 mm, and 0.38 mm for the group with normal, moderate, and bad bone quality, respectively. At 500 N force, the average displacements were 1.03 mm, 1.01 mm, and 1.86 mm and at 1000 N force, the average displacements were 1.88 mm, 2.11 mm, and 2.79 mm. Table 2 provides the main results for the individual specimens and the averaged values for the three bone quality groups.

**TABLE 2:** Look-up table for bone density as measured in CT, the evaluated bone quality, the length of the implanted screw, the percentage of screw length and projected screw surface area in the pedicle region, the percentage of resistance in the pedicle region as an absolute value and corrected for the projected screw surface area, the displacement values and the stress values in the pedicle and corpus region for 100 N, 500 N, and 1000 N are listed for every specimen and as averages for the three bone quality groups (HU = Hounsfield unit, PSS = projected screw surface).

Sample		Bone characteristics		Screw length	Screw in pedicle region		Resistance of pedicle region (absolute)			Resistance of pedicle region (relative to area)			Screw displacement [mm]			Stress at 100 N [MPa]		Stress at 500 N [MPa]		Stress at 1000 N[MPa]	
#	Side	Density [HU]	Quality	[mm]	Length (%)	PSS (%)	100 N	500 N	1000 N	100 N	500 N	1000 N	100 N	500 N	1000 N	Corpus	Pedicle	Corpus	Pedicle	Corpus	Pedicle
1	Right	189	Normal	42	62	66	40%	58%	63%	0.61	0.87	0.95	0.24	0.74	1.16	0.90	0.31	3.20	2.23	5.60	4.88
1	Left	189	Normal	42	67	71	67%	85%	89%	0.94	1.19	1.26	0.26	0.85	1.21	0.58	0.48	1.36	3.05	1.89	6.43
2	Right	172	Normal	36	61	66	55%	53%	77%	0.83	0.80	1.17	0.23	0.92	2.57	0.81	0.50	4.19	2.42	4.10	7.05
2	Left	172	Normal	42	62	66	49%	51%	68%	0.74	0.78	1.02	0.30	1.26	2.08	0.77	0.38	3.69	1.99	4.89	5.24
3	Right	120	Normal	42	62	66	55%	66%	70%	0.83	1.00	1.05	0.22	1.25	2.29	0.68	0.43	2.57	2.56	4.59	5.40
3	Left	120	Normal	48	67	71	65%	43%	58%	0.92	0.60	0.82	0.16	1.15	1.96	0.53	0.41	4.35	1.34	6.42	3.63
4	Right	117	Moderate	30	60	66	50%	68%	77%	0.76	1.02	1.16	0.42	1.41	2.06	1.08	0.56	3.52	3.78	5.09	8.56
4	Left	117	Moderate	30	80	88	80%	82%	87%	0.91	0.94	0.99	0.46	1.32	2.98	1.20	0.67	5.36	3.46	7.86	7.31
5	Right	113	Moderate	42	48	51	42%	43%	63%	0.82	0.84	1.23	0.16	0.92	2.57	0.61	0.42	3.00	2.14	3.88	6.32
5	Left	113	Moderate	42	62	66	60%	61%	76%	0.91	0.92	1.14	0.09	0.39	0.84	0.61	0.46	2.94	2.37	3.71	5.85
6	Right	90	Bad	46	43	46	41%	77%	85%	0.88	1.67	1.84	0.16	2.08	3.63	0.51	0.41	0.99	3.88	1.30	8.55
6	Left	90	Bad	44	41	44	47%	40%	51%	1.07	0.91	1.17	0.11	1.03	1.79	0.46	0.52	2.60	2.23	4.22	5.73
7	Right	1	Bad	36	72	78	75%	88%	95%	0.96	1.13	1.21	0.16	1.47	2.44	0.68	0.58	1.67	3.40	1.43	7.34
7	Left	1	Bad	36	50	54	67%	87%	94%	1.25	1.62	1.75	1.08	2.85	3.31	0.43	0.75	0.84	4.88	0.74	10.56
Mean for normal bone quality				42	63	68	55%	59%	71%	0.81	0.87	1.04	0.24	1.03	1.88	0.71	0.42	3.23	2.26	4.58	5.44
Mean for moderate bone quality				36	62	68	58%	63%	75%	0.85	0.93	1.13	0.28	1.01	2.11	0.87	0.53	3.70	2.94	5.13	7.01
Mean for bad bone quality				41	52	55	58%	73%	81%	1.04	1.33	1.49	0.38	1.86	2.79	0.52	0.57	1.52	3.60	1.92	8.05

## Discussion

Pedicle screw loosening poses a relevant complication risk after posterior instrumentation of the lumbar spine. Consequently, the aim of this study was to qualitatively analyze the local support of the vertebral body along traditional pedicle screw trajectories in the caudal direction, and to quantify the contribution of the pedicle and corpus region in relation to bone quality and loading amplitude.

The overlays of the stress-displacement plots with the parasagittal micro-CT reconstructions along the screw trajectories (Figures 4–6) illustrate the local resistance of the vertebral bodies against craniocaudal loading acting on different regions of the screw. With maximal stresses of roughly 20 MPa during testing, all of the imprint tests reached the cortical shell or the endplate of the vertebral bodies. Because the ultimate strength of cancellous bone in vertebral bodies ranges from 0.6 to 7.8 MPa (Mosekilde and Mosekilde, 1986; Banse et al., 2002), this finding is in line with the expected behavior of cancellous bone under such loading conditions.

The overall shapes of the load-deflection curves can be grouped into roughly three types of behavior: curves with a rather constant stress absorbance (plateau phase) throughout a large portion of cancellous bone after an initial ramping phase, curves with a slowly progressive stress absorbance in cancellous bone after a similar initial ramping phase and finally, curves with a rather sharp increase in stress absorbance until the end of the imprint test. The first two curve types are typically found in the corpus region and the third type is primarily seen in the pedicle region. Interestingly, in situations with a large enough gap between the screw and the cortical shell, a similar plateau phase can also be seen in measurements of the pedicle region (e.g., #1, right). The stress values at the plateau phase are assumed to be closely related to the local ultimate strength because further displacement can only be achieved through local failure of the trabecular bone structures. Therefore, the specimens with a large plateau phase in the corpus region provide a rather straight-forward method to compare the stress resistance of trabecular bone in relation to bone quality. In specimens with normal bone quality, the plateau phase varies in the region of roughly 5 MPa, while in specimens with bad bone quality the plateau phase ranges at values from 1 to 3 MPa. Although general trend of lower stress absorbance in situations with reduced bone quality can clearly be seen in the presented results, some notable exceptions must be discussed: For example, the corpus region of the left side of specimen #6 shows high local bone density (as seen in the micro-CT image) and, despite the overall bone quality being labeled as bad in this specimen, the local stress resistance in this area is surprisingly good with values exceeding 10 MPa (Figure 6). In contrast, the area of the tip piece of the right side of specimen #4 resists only 1–2 MPa, even though the measured HU units of this specimen are just below the defined cut-off value for normal bone quality (117 HU, while above 120 HU normal bone quality is assumed). While these two examples demonstrate the importance of local bone density for screw hold, the general trend observed in the results with higher resistance with higher HU-units nicely shows the informative value of the rather simple and clinically applicable method to evaluate bone quality (Schreiber et al., 2011).

In the final 1–2 mm of the imprint tests, a rather steep increase in stress absorbance can be seen in virtually all measurements, independent of the location along the screw trajectory and largely unaffected by the previous shape of the load-deflection curve. This rather sharp increase in resistance is interpreted as the result of (trabecular) bone material compaction against the stable cortical shell or vertebral endplate, as well as the known increase of bone mineral density towards the cortical shell in the pedicle region (Hirano et al., 1997). In situations of minimal distance between screw and cortical shell (e.g., pedicle region of #1 left), only very small displacement values are needed to meet large resistance. This finding nicely illustrates the benefits of placing a pedicle screw close to the cortical shell which can be achieved by increasing screw diameter (larger pedicle fill) or by choosing a specific trajectory such as CBT. Another approach to achieve firm contact with the cortical shell of the pedicle region could be to use bone cement augmentation in the pedicle region. Cement augmentation of the pedicle region would further provide the advantage of reducing the potential risk of iatrogenic pedicle fracture due to the insertion of an oversized pedicle screw, and bone cement could fill the pedicle independent of its anatomical shape.

To compare the contribution of the corpus and the pedicle region quantitatively, the behavior at the measured locations was used to interpolate the missing locations along the screw, which allowed us to virtually displace the whole screw caudally and compute the relative contribution of the corpus and pedicle region, the mean stresses at the screw-bone interface of these two regions, as well as the screw’s displacement.

In the situation of low loading amplitudes (100 N acting on the whole screw), no failure at the screw-bone interface is expected and differences in cortical bone stiffness can be assumed to be the primary factor for the local differences in screw support. In this situation, the contribution of the pedicle and corpus region are largely balanced, with the pedicle region providing an average of 55%–58% for all three bone quality groups (Figure 7). Interestingly, because the projected screw surface area in the pedicle is larger than the screw surface area in the corpus, the local stress resistance of the pedicle region is roughly 20% smaller compared to the corpus region in specimens with normal and moderate bone quality. In other words, the trabecular bone of the corpus provides better support than the trabecular bone of the pedicle at low loading amplitudes in this data. This finding appears contra intuitive because bone mineral density, which is known to be associated with trabecular bone strength (Keller, 1994), is typically larger at the pedicle region compared to the corpus region (Hirano et al., 1997). Furthermore, the screw’s surface is closer to the cortical shell in the pedicle region, which could provide better support (and less compliance) compared to the situation in the corpus with the vertebral endplate being much further from the screw surface. One potential explanation for this finding is the consideration of the predominant loading directions of the two regions. While the primary loading direction in the corpus is craniocaudal resulting in a predominant vertical orientation of the trabecula (Bartel et al., 2006), more diverse loading directions can be postulated in the pedicle region. With the known anisotropy of trabecular bone (Bartel et al., 2006), and following Wolff’s law of bone adaptation in relation to the acting stresses, the microstructure of the corpus could be better suited to counter the craniocaudally oriented loading of the screw compared to the trabecular bone of the pedicle region. Currently, much effort is invested to improve screw fixation strength by optimizing screw trajectories according to the local distribution of bone mineral density. Considering the hypothesized local differences in anisotropy of the trabecular bone in the corpus and trabecular region could help to further optimize this approach, and could even serve as the basis to develop novel implant designs to benefit from this effect.

With intermediate loading amplitudes (500 N acting on the whole screw), local failure can occur, and therefore ultimate strength of the bone can be assumed to play a more important role compared to the previously discussed loading situation. While a small increase in the support provided by the pedicle region can be observed in specimens with normal and moderate bone quality, this increase is much more accentuated in specimens with bad bone quality (Figure 7). In addition, the screw displacement in specimens with bad bone quality is more than twice as large compared to the specimens with normal or moderate bone quality. The average screw displacement of 1.86 mm in specimens with bad bone quality could potentially result in screw loosening because a radiolucency of more than 1 mm is often used as a radiographic criterion to diagnose screw loosening (Galbusera et al., 2015). As previously discussed, one way to approach this problem is to place the pedicle screw closer to the cortical shell, by choosing a larger screw diameter (resulting in a smaller gap to the cortical shell), or by augmenting the trabecular bone of the pedicle with bone cement. With these measures, the potential screw displacement (until sufficient resistance is provided) is reduced, which could be beneficial in the prevention of screw loosening.

In the high loading amplitude situation (1000 N acting on the screw), the trends observed at the intermediate loading situation are emphasized and the relative contribution of the pedicle region is further increased to 71%, 75%, and 81%. This can be explained by the stress resistance of the pedicle region being larger than the stress resistance of the corpus region in this loading scenario. This constellation stands in contrast to the situation with 100 N loading and highlights the importance of the pedicle region for such (unphysiologically) high loading amplitudes. The load redistribution can further be seen in the changes of the local stresses from 500 N to 1000 N. At the corpus region, only a relatively small increase of the local stress can be seen (averaging at +35%), while the average increase in the pedicle region is +140%.

This work has experienced several limitations. First, the chosen loading condition is a gross simplification of the complex loading conditions that can occur during activities of daily living and represents just one specific loading scenario in which the pedicle screw is loaded perpendicular to its axis in a caudal direction. Additional loading conditions, such as bending or pull-out forces, are not represented and the consequence of different compliance along the screw axis is not included. Furthermore, simple load-to-failure tests were performed and parameters such as fatigue failure or biological adaptation such as the bone remodeling are not considered. The method that was used to interpolate the measurement data to the whole screw is associated with some uncertainty and could interfere with the results. Furthermore, screw insertion depth and with that the distribution of the projected screw surface in the corpus and pedicle region were not standardized, and therefore some variability exists between specimens (Table 2). Because the averaged values were not largely different between the groups, the effect on the data analysis should be acceptable. Nevertheless, the authors believe that thanks to this simple approach, the gained insights can be well understood and help to further our understanding of the problem of screw loosening.

Replica screw insertion and bandsaw cutting of the specimens could have induced some damage to the vertebral bodies, which could have in turn affected the measurements. Visually, there were no signs of relevant bone damage due to the specimen preparation. Because any potential damage would be affecting all measurement locations to a similar degree, the effect on the results should be minimal. To achieve reliable specimen fixation, PMMA-potting was used, which can generate elevated temperatures due to the exothermic nature of the chemical reaction (Amin et al., 2015). While these elevated temperatures might harm biological tissue, the effect on the mineralized bone material is evaluated to be of minor importance. The size of the screw pieces used for imprint testing was chosen at 6 mm to provide sufficient surface area to be more robust against very localized differences in bone density. Nevertheless, with the sharp edge at the performed cut, the failure mechanism of trabecular bone could be different from an intact pedicle screw without such edges. The distance between imprint tests was chosen at 6 mm to limit the effect on the adjacent segments. Furthermore, the testing sequence was reversed for half of the specimens to limit any systematic effect. Nevertheless, some effect on the results cannot be excluded. Based on the experimental work by Grant et al., the ultimate strength of the inferior endplate of lumbar vertebrae can be assumed to be roughly 10–15 MPa (assuming 100–150 N failure load with the indenter of 3 mm diameter) (Grant et al., 2001). Bone mineral density of the cortical shell of the pedicle region can be assumed to be at around 800 mg/cm^3^ (Hirano et al., 1997), which would correspond to an ultimate strength of roughly 70 MPa (Keller, 1994; Schileo et al., 2008). This is in line with ultimate strength values for human cortical bone reported in the literature (Mirzaali et al., 2016; Wolfram and Schwiedrzik, 2016). Therefore, the cortical shell of the pedicle should be able to withstand the applied stresses; however, endplate failure or cortical shell breakthrough could occur. The absence of such failure in our data must be ascribed to the additional support given by the PMMA-embedment. Measurements with stresses exceeding the expected failure level must therefore be analyzed with prudence because the resistance of the endplate could be overestimated. Nevertheless, the increase in stress absorbance towards the endplate is assumed to be representative of reality, while the final peak values might be too high. Given that in all virtual loading scenarios (100 N, 500 N, and 1,000 N acting on the screw) the maximal stress values were below 11 MPa, this analysis should not be affected.

## Conclusion

Unidirectional imprint tests of pedicle screw sections perpendicular to the screw surface have been performed to analyze the resistance along the pedicle screws following the traditional trajectory. At low loading amplitudes, the trabecular bone of the corpus region appears to provide slightly more support than the trabecular bone of the pedicle region. This observation could be the result of trabecular bone anisotropy, which could be used to further optimize screw trajectories and implant designs. At higher loading amplitudes, and especially in specimens with reduced bone quality, the contribution of the pedicle region becomes predominant, which can be ascribed to the increasing support of the cortical shell after some screw displacement towards the cortex.

To reduce the risk of screw loosening, it could be beneficial to limit the available subsidence distance until adequate resistance is met. To achieve this goal, the distance to the cortical bone could be reduced by placing the pedicle screw closer to the cortex, by selecting a larger screw diameter, or potentially by augmenting the pedicle with bone cement.

## Data Availability

The original contributions presented in the study are included in the article/Supplementary Material, and further inquiries can be directed to the corresponding author.
